# Experimental Evidence of Amplitude Death and Phase-Flip Bifurcation between In-Phase and Anti-Phase Synchronization

**DOI:** 10.1038/s41598-018-30026-3

**Published:** 2018-08-02

**Authors:** Krishna Manoj, Samadhan A. Pawar, R. I. Sujith

**Affiliations:** Indian Institute of Technology Madras, Chennai, 600036 India

## Abstract

Nonlinear phenomena emerging from the coupled behaviour of a pair of oscillators have attracted considerable research attention over the years, of which, amplitude death (AD) and phase-flip bifurcation (PFB) are two noteworthy examples. Although theoretical research has postulated the coexistence of AD and PFB upon variation of different control parameters, such an occurrence has not been reported in practical systems. Here, we provide the first experimental evidence of the coexistence of AD and PFB in a physical system, comprising of a coupled pair of candle-flame oscillators. As the strength of coupling between the oscillators is increased, we report a decrease in the span of AD region between the states of in-phase and anti-phase oscillations, leading up to a point of PFB. Understanding such a switching of phenomena between AD and PFB helps us to evade their undesirable occurrences such as AD in neuron and brain cells, oscillatory state in prey-predator systems, oscillatory spread of epidemics and so forth.

## Introduction

System of coupled oscillators has become an extensive area of research due to the emergence of various intriguing nonlinear phenomena^1–4^. Mutual coupling between oscillators can give rise to synchronization^4^ and amplitude death^5^ (AD). Synchronization is a universal phenomenon characterized by an adjustment of rhythms of coupled oscillators, whereas AD is the cessation of their oscillations. The transition from one synchronization state to another can occur gradually through an intermediate state of AD^6^ or with a sudden shift known as phase-flip bifurcation^7^ (PFB). To elaborate, during PFB, oscillators exhibit a sudden shift in their synchronized state from in-phase (IP) to anti-phase (AP) along with a jump in their frequencies^8^. Such coupled behaviours are of importance in various physical, biological, chemical, ecological, and engineering systems^9–13^. Although most of the theoretical studies indicate the individual or combined occurrence of AD and PFB^5,14–17^, an experimental evidence of their coexistence in a physical system has not been reported to the best of our knowledge.

**Figure 1:**
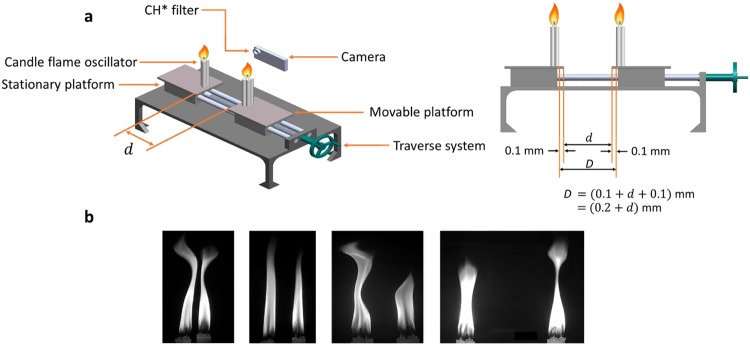
Experiment setup and flame images corresponding to different modes of coupled behaviour. **(a)**, Schematic of the experimental setup (isometric and side view) used for studying the coupled behaviour of a pair of candle-flame oscillators by varying the distance between them. One oscillator is mounted on a stationary platform and the other on a movable platform. The latter is moved with the help of a traverse system, thereby varying the distance ($d$) between them, which in turn, varies the edge to edge distance between the oscillators ($D$). High speed images of heat release rate fluctuations, at each value of $d$, were acquired using a camera with a CH* chemiluminescence filter mounted in front of it. Similar experiments were conducted by varying the number of candles in each oscillator from 3 to 8. **(b)**, Snapshots of the flame images as captured at characteristic distances between the oscillators highlighting the oscillatory states of IP ($d\,$= 0 cm), AD ($d\,$= 1 cm), AP ($d$ = 2 cm) and desynchronization ($d$ = 7 cm), respectively. A detailed description of the experimental setup and the data analysis are provided in Methods. We acknowledge Mr. Thilagraj, Junior Technician in the Department of Aerospace Engineering, Indian Institute of Technology Madras, for providing the schematic.

Here, we show the coexistence of AD and PFB for the first time in a physical system. The system presented in our study comprises of coupled candle-flame oscillators (refer Fig. 1), whose synchronization has recently become a topic of immense interest^18–20^. The basic requirement for synchronization is the presence of self-sustained oscillators and the coupling between them^4^. Since such self-sustained oscillations are not exhibited by a single candle flame (which only shows transient fluctuations called flickering), a set of 3 or more candles, referred to as candle-flame oscillator, are ignited to form a single flame which exhibits self-sustained limit cycle oscillations^18,21^. Studies on mutual coupling between a pair of such oscillators have so far reported the presence of IP and AP states alone, when the distance between them is varied^18^. In the present study, we explore the transition from IP to AP in candle-flame oscillators, which can occur gradually through an AD state or by PFB. To demonstrate such transitions, control parameters such as the distance between the oscillators (*d*) and the number of candles in each oscillator (*N*_*C*_) are varied. Varying *d* serves as a mechanism to change the time delay between the oscillators, whereas *N*_*C*_ aids in changing their amplitudes of oscillations and thereby their coupling strength. The transition with the presence of AD is described here in candle-flame oscillators consisting of four candles each.

## Results

**Figure 2:**
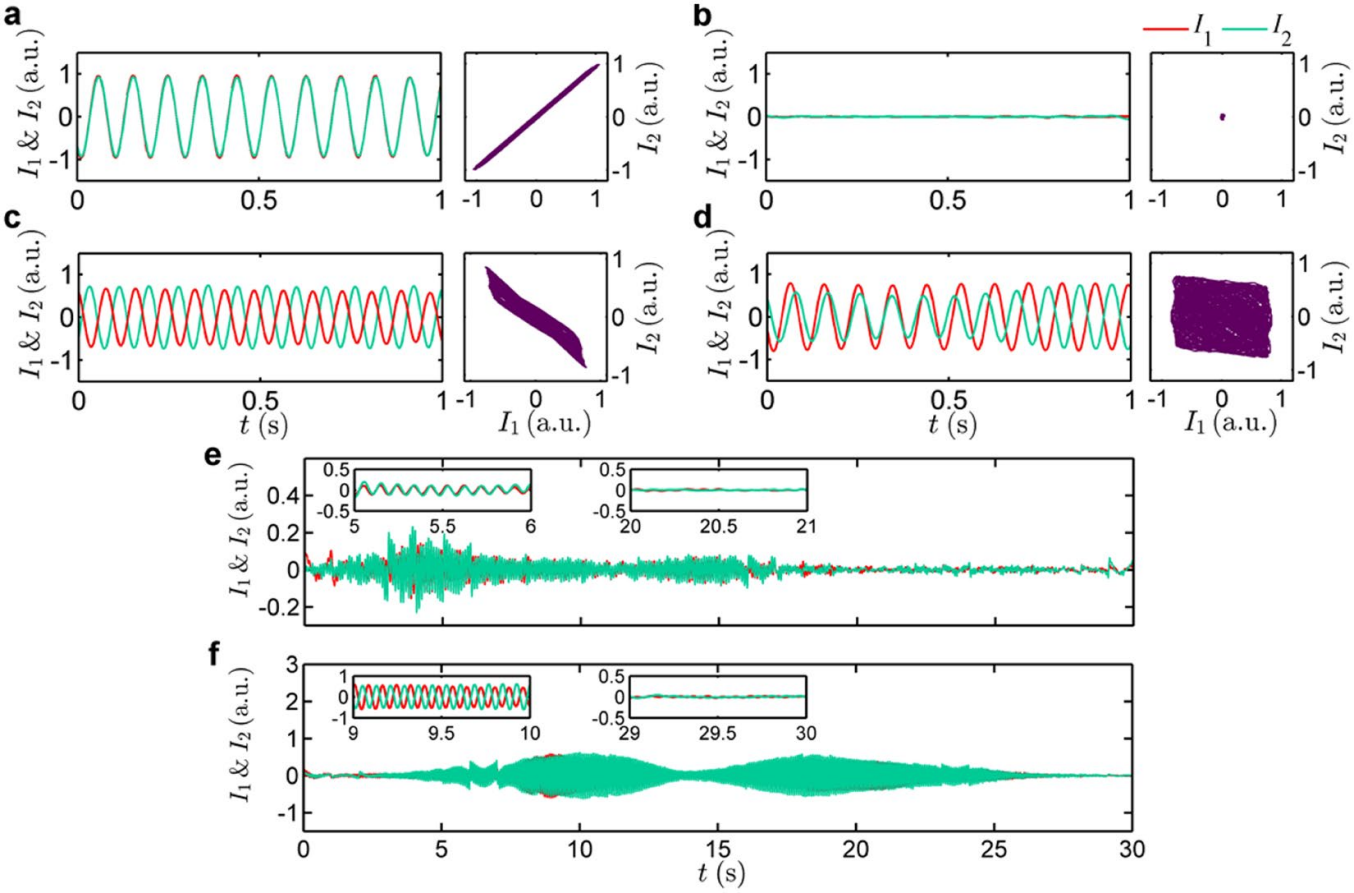
Dynamics of a pair of candle-flame oscillators representing various states of coupled behaviour observed at different values of $d$. (**a–d**), Time series of heat release rate fluctuations ($\,{I}_{1}$ and ${I}_{2}$) obtained from individual oscillators and their corresponding amplitude correlation plot (a plot between ${I}_{1}$ and ${I}_{2}$) are shown for IP, AD, AP, and desynchronized state of oscillations, respectively. These states are observed when the value of $d$ is 0 cm, 1 cm, 2 cm, and 7 cm, respectively. ${I}_{1}$ and ${I}_{2}\,\,$correspond to the instantaneous values of the heat release rate fluctuations of the oscillator on the stationary platform and the movable platform, respectively. (**e,f**), The transition states observed in a pair of candle-flame oscillators when the oscillators are 0.5 cm and 1.5 cm apart, respectively. In (**e)**, the time-series oscillates alternately between IP and AD states, while in (**f**), the oscillations alter between AD and AP states.

Figure 2 shows the dynamical states manifesting IP, AD, AP and desynchronized oscillations observed due to mutual interaction between the candle-flame oscillators over various values of *d*. At small values of *d* (0 cm to 1 cm), they exhibit IP synchronization (see Supplementary Videos 1 and 2), during which the time series of each oscillator (*I*_1_ and *I*_2_) fluctuates with nearly 0 deg phase difference (Fig. 2a). The collapse in the amplitude correlation plot (*I*_1_ against *I*_2_) into a line having a slope of positive unity (Fig. 2a) reasserts the presence of IP state. When *d* is increased beyond 1 cm, we notice a cessation of oscillations in both oscillators (Fig. 2b). We refer to this state as AD (see Supplementary Videos 3 and 4). The cluster of trajectory around the origin in the amplitude correlation plot implies the presence of AD in the signal (Fig. 2b). When *d* is sufficiently high (2 cm to 5 cm), both oscillators regain their oscillatory behaviour. However, at these distances both the oscillators exhibit AP synchronization (see Supplementary Videos 5 and 6), wherein the time series of each oscillator shows a phase difference of nearly 180 deg (Fig. 2c). Collapsing of the amplitude correlation plot into a line with a slope close to negative unity (Fig. 2c) reaffirms the AP synchronization of these oscillators. As *d* is increased further (beyond 5 cm), we observe desynchronization (DS) (see Supplementary Videos 7 and 8) of coupled candle-flame oscillators. At this state, we see an increased presence of IP oscillations in between AP oscillations in an arbitrary manner (Fig. 2d). This increased switching between IP and AP is further reflected in the amplitude correlation plot, wherein the plot fills the entire plane (Fig. 2d).

Apart from these steady states observed at particular ranges of *d* (shown in Fig. 2a–d), we also see transition states (TS) at the boundaries of AD. Figure 2e,f show the existence of such states in the transition from IP to AD, and AD to AP, respectively. The silent region represents AD, whereas the bursts show IP oscillations in Fig. 2e (see Supplementary Video 9) and AP oscillations in Fig. 2f (see Supplementary Video 10). The evidence of such transition states further reasserts that the transition from IP to AD and that from AD to AP is not sudden, but happens gradually.

**Figure 3:**
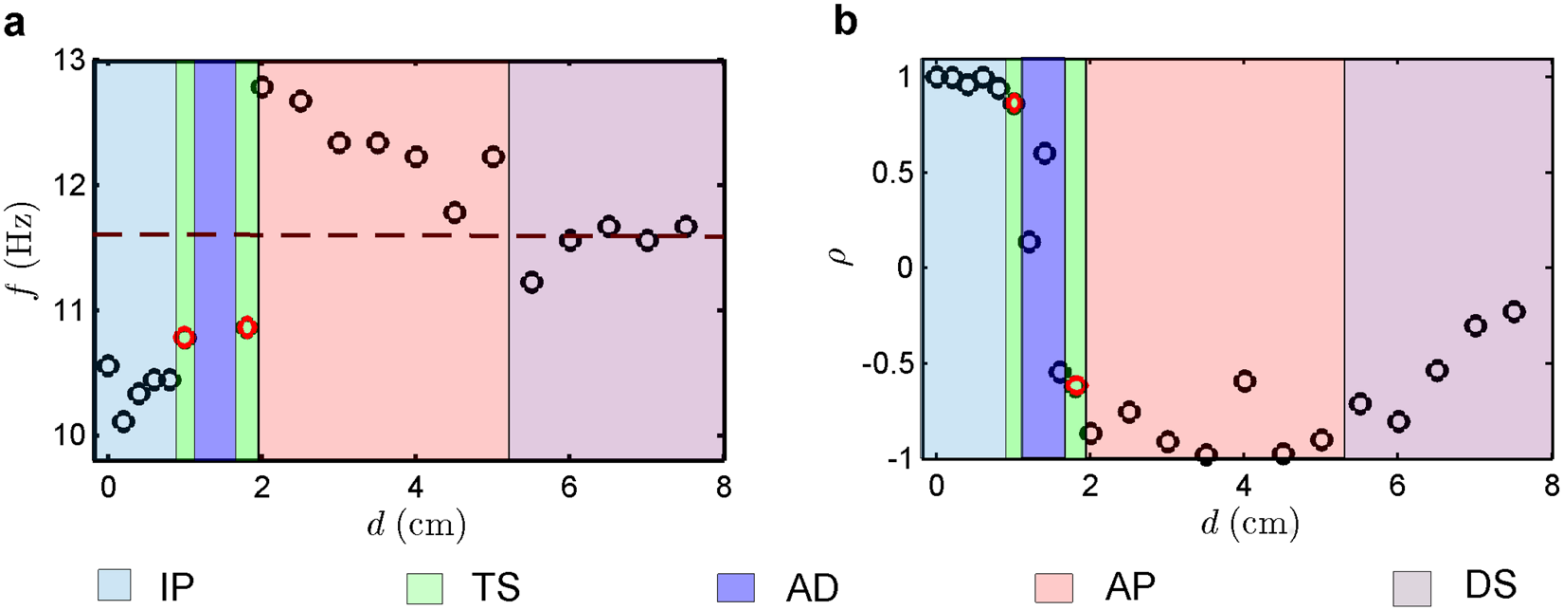
Characterization of synchronization transitions in coupled candle-flame oscillators due to variation in $d$. The variation of **(a)**, dominant frequency ($f$) and **(b)**, Pearson’s correlation coefficient ($\rho$) as a function of $d$ for a pair of candle-flame oscillators. The horizontal dotted line in (**a)** corresponds to the dominant frequency of 11.6 Hz obtained from the individual oscillator in an uncoupled state. IP oscillations can be characterized by the drop in the frequency value as well as $\rho$ tending to positive unity. In contrast, AP oscillations are characterized by an increase in the frequency value when compared to the uncoupled oscillator as well as $\rho$ tending to negative unity. The transition from IP to AP in the frequency plot (in **a**) is interposed by the region of AD state. The red circles correspond to the transition states (TS) where the frequency peak in the amplitude spectra represents the oscillatory regions observed in the signals of these states. As the oscillators move to desynchrony, the frequency fluctuates around the frequency of uncoupled oscillators along with $\rho$ moving towards 0, highlighting the reduction in interaction between the oscillators. The amplitude variation of the signals from both oscillators, obtained in terms of root mean square value, as a function of $d$ is provided in Supplementary Fig. 1.

Furthermore, we quantify the synchronization behaviour of candle-flame oscillators with change in *d* using various quantitative measures such as dominant frequency (*f*) of oscillations (Fig. 3a) and Pearson’s correlation coefficient (*ρ*) between the signals (Fig. 3b). When two oscillators are in IP state (Fig. 3a), we observe that the dominant frequencies of both the oscillators are below their uncoupled frequency value. As these oscillators transition from IP to AP via AD state, we notice a sudden jump in the values of their dominant frequencies (Fig. 3a) to a value greater than their uncoupled frequency. A possible explanation for this variation in frequencies is the change in the interaction of vortices formed due to the instabilities of buoyancy-driven flows around the oscillating flame of each candle-flame oscillator^20^. When the oscillators are nearby, the inner portions of the vortices merge with each other resulting in the inhibition of oscillations and thereby a reduction in their frequencies. On the contrary, when the oscillators are sufficiently far apart, alternate formation of such vortices enhances the oscillation in each oscillator leading to an increase in their natural oscillation frequency. During the state of AD (Fig. 3a), we do not observe any dominant peak in the amplitude spectra of both the oscillators, due to the complete disappearance of vortex formation in both the oscillators. However, we see the presence of oscillations during the transition states, which correspond to the two points in the TS zone of Fig. 3a, observed at the boundaries of AD. As *d* is further increased, we observe a decrease in the frequencies of both the oscillators due to a reduction in the interaction between them, finally reaching the value of frequency of an isolated oscillator in the DS region (Fig. 3a).

This synchronization behaviour is also reflected in the plot of the Pearson’s correlation coefficient (*ρ*) (Fig. 3b). If the two oscillators are in the IP state, the slope in the amplitude correlation plot tends to positive unity (Fig. 2a), so does the value of *ρ* (Fig. 3b). Conversely, for the AP state, we see a slope which tends to negative unity (Fig. 2c) and the value of *ρ* falls near minus one (Fig. 3b). When the oscillators are in the desynchronized state (Fig. 2d), the value of *ρ* approaches zero (Fig. 3b).

**Figure 4:**
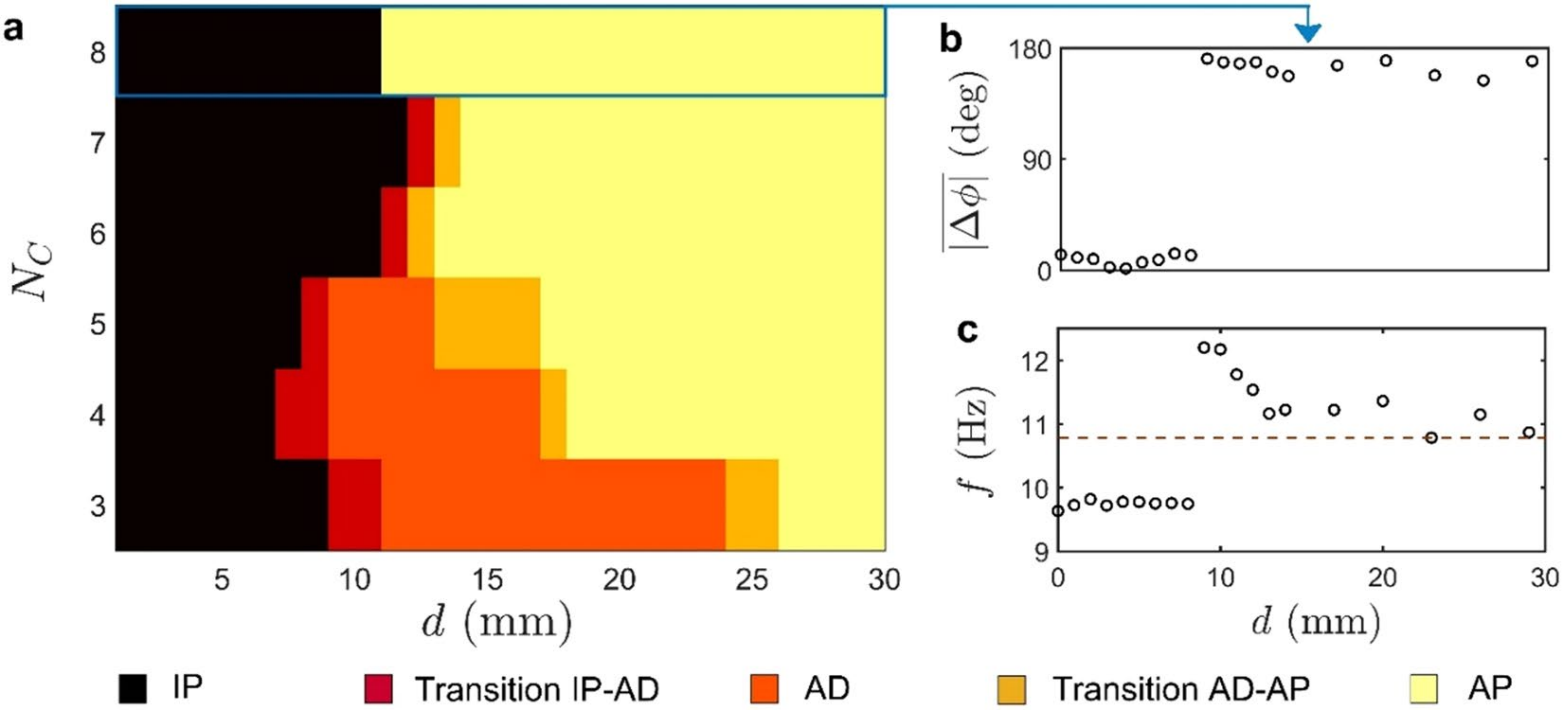
A two-parameter bifurcation plot between ${{\boldsymbol{N}}}_{{\boldsymbol{C}}}$ and ${\boldsymbol{d}}$ depicting the coexistence of AD and PFB in the dynamics of coupled candle-flame oscillators. **(a)** The mapping of the occurrence of various states of coupled dynamics in a pair of candle-flame oscillators with variation in two control parameters, $d$ and ${N}_{C}$. ${N}_{C}$ is varied from 3 to 8, while $d$ is increased from 0 to 30 mm in steps of 1 mm. (**b**,**c**) The variation in the  time averaged value of absolute relative phase and the dominant frequency, respectively, with $d$ for a pair of candle-flame oscillators consisting of ${N}_{c}$ = 8, highlights the phenomena of PFB. With increase in ${N}_{C}$, the region of AD gradually reduces to zero leading to the onset of PFB in the parameter space of $d$. The dominance of the AD or PFB exhibited by the system is decided by the control parameter ${N}_{C}$. At lower $\,{N}_{C}$, AD dominates whereas at higher ${N}_{C}$, PFB prevails. These observations of PFB along with AD in the same physical system of coupled candle-flame oscillators can be qualitatively replicated in a theoretical model of delay coupled identical Stuart-Landau oscillators (refer for further information to Supplementary Note 1 and Supplementary Figs. 3–5).

Finally, we investigate the effect of variation in the number of candles (*N*_*C*_) in candle-flame oscillators. Increase in *N*_*C*_, from 3 to 8, in an isolated oscillator leads to an increase in the amplitude and a small change in the frequency of oscillations (see Supplementary Fig. 2 and Supplementary Video 11). Figure 4 demonstrates the effect of variation in the number of candles (*N*_*C*_) in a pair of candle-flame oscillators. From Fig. 4a, we observe that the regions of AD (including both AD and transition states) are observed to decrease with increasing *N*_*C*_. At lower *N*_*C*_ (e.g. 3, 4 and 5), AD is observed over a considerable interval of *d*, whereas at higher *N*_*C*_ (e.g. 6 and 7), the likeliness of observing perfect AD becomes zero and only regions of transition states are observed. As *N*_*C*_ is further increased to 8, we observe the phenomenon of phase-flip bifurcation (PFB), where the oscillators exhibit a sudden transition from a state of IP to AP oscillations, bypassing the intermediary AD state. During this transition, we observe a sudden jump in the relative phase between the oscillators from near 0 deg to 180 deg, at the bifurcation point (Fig. 4b). In addition to this, while in IP state, we also observe a reduction in the frequency of both the oscillators from the value at their uncoupled state, followed by an abrupt jump in the frequency during the onset of AP state^7^ (Fig. 4c).

With increasing *N*_*C*_, we observe a reduction in the AD region along with an advancement in the onset of AP oscillations (Fig. 4a). We conjecture that with increasing *N*_*C*_, the amplitude of oscillations of individual oscillators increases due to insufficient air supply (see Supplementary Fig. 2), which in turn, increases the coupling strength between them. The enhancement in amplitude of the candle-flame oscillations reduces the possibility of displaying AD and develops a tendency in the coupled oscillators to move to a state of higher frequency (AP state, see Fig. 3a) so as to satisfy their oxygen needs.

## Discussion

Here, we examine the coupled interaction of candle-flame oscillators by varying two system parameters (*d* and *N*_*C*_). We demonstrate the experimental observation of the presence of AD in between IP and AP states along with PFB in the same physical system. AD can be achieved by various types of coupling such as diffusive, time-delay, conjugate and dynamic^5^. Among these, AD due to time-delay^6,9^ and indirect coupling (conjugate, relay, environmental diffusive)^16,17,22^ are often observed in physical systems, for instance, biological or chemical oscillators. Time-delay is intrinsic in such systems, as a finite amount of propagation time is required for transmission of the signal from one oscillator to another. In contrast, during indirect coupling, the oscillators are coupled through an intermediate medium (environment). Recent studies^7,16,22,23^ have shown that these types of couplings are also responsible for the manifestation of PFB. Since time-delay and indirect (through intermediate medium) couplings are the most important factors responsible for the demonstration of PFB along with AD^7,16,17^, we hypothesize that these coupling mechanisms could play a major role in the synchronization of coupled candle-flame oscillators as well. Such couplings are inherent in the oscillatory combustion of candle flames due to the difference in parameters such as the vaporization rate of wax, the chemical kinetics, and the time required for propagation of the thermal wave from one oscillator to another through a medium of oscillatory convecting current^24^. The increase in *d* further induces a delay in the coupling of these oscillators, which in turn, is responsible for the demonstration of different modes of synchronization.

The coexistence of AD and PFB allows us to vary the appropriate control parameters in order to choose the most desired phenomena amongst the two. The situation of AD is undesirable in many biological systems, hence a change in a single parameter which helps us to bypass this situation by directly moving to a state of PFB might act as a solution to many non-curable diseases such as Alzheimer’s and Parkinson’s disease^25,26^. On the contrary, AD is preferred (due to its remarkable stability) in ecological systems^27^, where an oscillatory phase would lead to the extinction of species in a long-term scenario. Thence, change in a control parameter leading to a switch from PFB to AD would help sustain various endangered species. The oscillatory transmission of measles epidemic in the United Kingdom in the 18^th^ century which persisted up to 20^th^ century was discovered to be IP at Birmingham and Newcastle, and AP at Cambridge and Norwich. The occurrence of AD state in such systems would have resulted in a drastic decrease in the spread of these diseases^28^. Furthermore, candle being a simple diffusion flame can also replicate phenomena observed in other large diffusion flames^29^. The application of coupled behaviour of oscillatory flames in a diffusion combustion system ranges from the propagation of natural fires resulting from accidents to human made combustion systems such as industrial, aircraft engine and rocket burners^19,30^.

## Methods

The candles used in the present experimental study were of cylindrical shape, made up of paraffin wax having a length of 15 cm and a diameter of 0.8 cm. A group of three or more such candles (*N*_*C*_ ≥ 3) were placed together in order to produce stronger self-sustained oscillations. These candles were tied to keep them intact and burnt after keeping their wicks close enough such that they burn to form a single flame that exhibits limit cycle oscillations. This set of candles is then referred to as a single candle-flame oscillator. We explore two scenarios; one in which *N*_*C*_ is same for both oscillators (similar pair) and another in which *N*_*C*_ is different in both oscillators (dissimilar pair). In the main text (Figs. 2 and 3), we present the results of synchronization behaviour of the former with each oscillator consisting of four candles.

The average frequency of natural oscillations of a single isolated candle-flame oscillator, each consisting of four candles, obtained from ten independent trial experiments is measured to be 11.6 Hz with a standard deviation of ± 0.3 Hz. The resolution of frequency in the amplitude spectrum of the signals is approximately equal to 0.1 Hz. The later part of our study uses coupled similar pair of oscillators with *N*_*C*_ varying from 3 to 8 (refer Fig. 4), whose isolated oscillator characteristics are shown in Supplementary Fig. 2. The second scenario of our study explores the dynamics of a coupled dissimilar pair of oscillators, and its results are discussed in Supplementary Figs. 6 and 7 and for more details refer to Supplementary Videos 12–15.

By moving the candle-flame oscillator placed on a movable platform against the stationary one, we vary the edge to edge distance (*D*) between them (Fig. 1a) which leads to a corresponding change in *d* (*D* = *d* + 0.02 cm). Here, *d* is varied from 0 cm to 2 cm in steps of 0.2 cm, and 2 cm to 8 cm in steps of 0.5 cm. The characteristic distance (*d*) at which various synchronization modes are observed may vary with ± 0.2 cm with experiments. An acrylic plate with hexagonal packing of drilled holes of 0.4 cm radius is fixed on each platform to ensure that the oscillators are held firmly in place. The traverse system, used to vary *d* by moving the platform, has a least count of 0.05 cm. The entire system is mounted on a raised platform (height 50 cm) to avoid ground effects on the dynamics of the candle-flame oscillations. All experiments were performed in a closed quiescent room with a completely dark environment.

High-speed imaging of the reaction zone of the flames^31^ from both the candle-flame oscillators is performed using a camera (Model-iPhone 6) having a CH* filter (wavelength 435 nm and 10 nm full width at half maximum) mounted in front of its lens. The data are acquired at 239 frames per second for 10 s for each *d* except during transitional states where the data are acquired for 30 s to have a broader view of the system dynamics. A waiting time of 20 s is enforced after each increment in *d*, to remove the transient effects and let the oscillators reach a steady state.

The analysis of the data set acquired at every value of *d* is performed after cropping the portion of each frame in such a way that no two flames of the oscillators appear in a single cropped frame. The local chemiluminescence intensity values of such frames are summed up so as to get a global instantaneous value of the heat release rate for each oscillator in a single video frame. Thus, a time series of such heat release rate fluctuations for each candle-flame oscillator is obtained separately.

The mutual synchronization features of two candle-flame oscillators due to change in the distance between them (*d*) are characterized by calculating the instantaneous phase difference between their signals^4^, as shown in Fig. 4b. To calculate the instantaneous phase of a signal, we use the analytic signal approach based on Hilbert transform. Hilbert transform derives an analytic representation of a time series signal, that is, the real signal is extended into the complex plane. The analytical signal (*ζ*(*t*)) thus obtained will have the original signal ($I(t)$) as its real part and its corresponding Hilbert transform (${I}_{H}(t)$) as its imaginary part (that is, $\zeta (t)=I(t)+i\,{I}_{H}(t)$). Here, ${I}_{H}(t)$ is given by

1 $${I}_{H}(t)=\,\frac{1}{\pi }P.V.{\int }_{-\infty }^{\infty }\frac{I(\tau )}{t-\tau }d\tau $$

where *P*.*V*. is the Cauchy principle value.

Therefore, the analytical signal thus obtained can be written in the form $\zeta (t)=A(t){e}^{i\varphi (t)}$, where $A(t)$ coresponds to the instanteneous amplitude and $\varphi (t)$ corresponds to the instantaneous phase of the signal. The relative phase ($\Delta {\varphi }_{1,2}$) between two signals is calculated from the difference in the instantaneous phases of these signals with time as follows^4^

2 $${\rm{\Delta }}{\varphi }_{1,2}(t)=\,{\varphi }_{2}(t)-\,{\varphi }_{1}(t)$$

and the condition for phase locking between the two signals^4^ is given by

3 $$|{\rm{\Delta }}{\varphi }_{1,2}(t)|\le constant$$

The synchronization in oscillations restricts the variation in the relative phase to remain bounded, thence exhibiting fluctuations around a constant value of the phase difference^4^. When the oscillators reach a state of desynchronization, the phase difference between them no longer remains bounded, a situation known as phase drifting^4^.

The mean value of the absolute phase angle between the signals of coupled oscillators can be calculated as

4 $$\overline{|\Delta \varphi |}=\frac{1}{N}\sum _{j=1}^{N}|{\rm{\Delta }}{\varphi }_{j}|$$

where *N* is the number of data points in the signal.

In order to calculate the amplitude correlation between the signals of coupled candle-flame oscillators, we use a measure of Pearson’s correlation coefficient^32^ (*ρ*). This measure quantifies the linear correlation between two signals (*I*_1_ and *I*_2_) of coupled oscillators (as shown in Fig. 3b). Its value lies in between + 1 and −1, which has direct correlation with the slope of the amplitude correlation plot (a plot between *I*_1_ and *I*_2_, as shown in Fig. 2). The value of the correlation coefficient is given by

5 $$\rho =\,\frac{N({\sum }^{}{I}_{1}{I}_{2})\,-(\,{\sum }^{}{I}_{1}{\sum }^{}{I}_{2})}{\sqrt{(N{\sum }^{}{I}_{1}^{2}-{({\sum }^{}{I}_{1})}^{2}\,)(N{\sum }^{}{I}_{2}^{2}-{({\sum }^{}{I}_{2})}^{2}\,)}}$$

where all summations are from $n=1$ to $N$.

## Electronic supplementary material

Supplementary Video 1

Supplementary Video 2

Supplementary Video 3

Supplementary Video 4

Supplementary Video 5

Supplementary Video 6

Supplementary Video 7

Supplementary Video 8

Supplementary Video 9

Supplementary Video 10

Supplementary Video 11

Supplementary Video 12

Supplementary Video 13

Supplementary Video 14

Supplementary Video 15

Supplementary Material

## References

[1] Winfree, A. T. *The Geometry of Biological Time* (Springer Science & Business Media, 2001).

[2] Kuramoto, Y. *Chemical Oscillations, Waves, and Turbulence* (Springer Science & Business Media, 2012).

[3] Strogatz, S. *Sync: The Emerging Science of Spontaneous Order* (Penguin UK 2004).

[4] Pikovsky, A., Rosenblum, M. & Kurths, J. *Synchronization: A Universal Concept in Nonlinear Sciences* (Cambridge university press, 2003).

[5] Saxena G, Prasad A, Ramaswamy R (2012). Amplitude death: The emergence of stationarity in coupled nonlinear systems. Phys. Rep..

[6] Reddy DR, Sen A, Johnston GL (1998). Time delay induced death in coupled limit cycle oscillators. Phys. Rev. Lett..

[7] Prasad A (2008). Universal occurrence of the phase-flip bifurcation in time-delay coupled systems. Chaos.

[8] Prasad A, Kurths J, Dana SK, Ramaswamy R (2006). Phase-flip bifurcation induced by time delay. Phys. Rev. E.

[9] Strogatz SH (1998). Nonlinear dynamics: Death by delay. Nature.

[10] Bar-Eli K (1985). On the stability of coupled chemical oscillators. Physica D.

[11] Blasius B, Huppert A, Stone L (1999). Complex dynamics and phase synchronization in spatially extended ecological systems. Nature.

[12] Reddy DR, Sen A, Johnston GL (2000). Experimental evidence of time-delay-induced death in coupled limit-cycle oscillators. Phys. Rev. Lett..

[13] Glass L (2001). Synchronization and rhythmic processes in physiology. Nature.

[14] Karnatak R, Punetha N, Prasad A, Ramaswamy R (2010). Nature of the phase-flip transition in the synchronized approach to amplitude death. Phys. Rev. E.

[15] Arumugam R, Dutta PS, Banerjee T (2016). Environmental coupling in ecosystems: From oscillation quenching to rhythmogenesis. Phys. Rev. E.

[16] Sharma A, Verma UK, Shrimali MD (2016). Phase-flip and oscillation-quenching-state transitions through environmental diffusive coupling. Phys. Rev. E.

[17] Karnatak R, Ramaswamy R, Prasad A (2007). Amplitude death in the absence of time delays in identical coupled oscillators. Phys. Rev. E.

[18] Kitahata H (2009). Oscillation and synchronization in the combustion of candles. J. Phys. Chem. A.

[19] Forrester DM (2015). Arrays of coupled chemical oscillators. Sci. Rep..

[20] Okamoto K, Kijima A, Umeno Y, Shima H (2016). Synchronization in flickering of three-coupled candle flames. Sci. Rep..

[21] Nagamine Y, Otaka K, Zuiki H, Miike H, Osa A (2017). Mechanism of Candle Flame Oscillation: Detection of Descending Flow above the Candle Flame. J. Phys. Soc. Jpn..

[22] Sharma A, Sharma PR, Shrimali MD (2012). Amplitude death in nonlinear oscillators with indirect coupling. Phys. Lett. A.

[23] Cruz JM (2010). Phase-flip transition in coupled electrochemical cells. Phys. Rev. E.

[24] McCaffrey, B. J. *Purely buoyant diffusion flames: Some experimental results. Final Report. Chemical and Physical Processes inCombustion. The National Institute of Standards and Technology (NIST)* 49 (1979).

[25] Mizuno Y (1989). Deficiencies in complex I subunits of the respiratory chain in Parkinson’s disease. Biochem. Biophys. Res. Comm..

[26] Lim AS (2017). Diurnal and seasonal molecular rhythms in human neocortex and their relation to Alzheimer’s disease. Nature Comm..

[27] Yoshida T (2003). Rapid evolution drives ecological dynamics in a predator-prey system. Nature.

[28] Duncan CJ, Duncan SR, Scott S (1997). The dynamics of measles epidemics. Theor. Popul. Biol..

[29] Faraday, M., *Faraday’s Chemical History of a Candle*. (Chicago Review Press, 1861).

[30] Yeoh, G. H. & Yuen, K. K. *Computational Fluid Dynamics in Fire Engineering: Theory, Modelling and Practice* (Butterworth-Heinemann, 2009).

[31] Hardalupas Y, Orain M (2004). Local measurements of the time-dependent heat release rate and equivalence ratio using chemiluminescent emission from a flame. Combust. Flame.

[32] Pearson KL (1901). On lines and planes of closest fit to systems of points in space. Lond. Edinb. Dubl. Phil. Mag..

