# Supplementary material for: Experimental Evidence of Amplitude Death and Phase-Flip Bifurcation between In-Phase and Anti-Phase Synchronization
Source: Sci Rep. 2018 Aug 2;8:11626. doi: 10.1038/s41598-018-30026-3 (PMC6072762; doi:10.1038/s41598-018-30026-3)
Supplement: Supplementary file 16 — Supplementary Material [file 41598_2018_30026_MOESM16_ESM.docx]

**Experimental Evidence of Amplitude Death and Phase-Flip Bifurcation between In-Phase and Anti-Phase Synchronization**

Krishna Manoj, Samadhan A. Pawar and R. I. Sujith*

Indian Institute of Technology Madras, Chennai 600036, India

Corresponding author: [sujith@iitm.ac.in*](mailto:sujith@iitm.ac.in*)


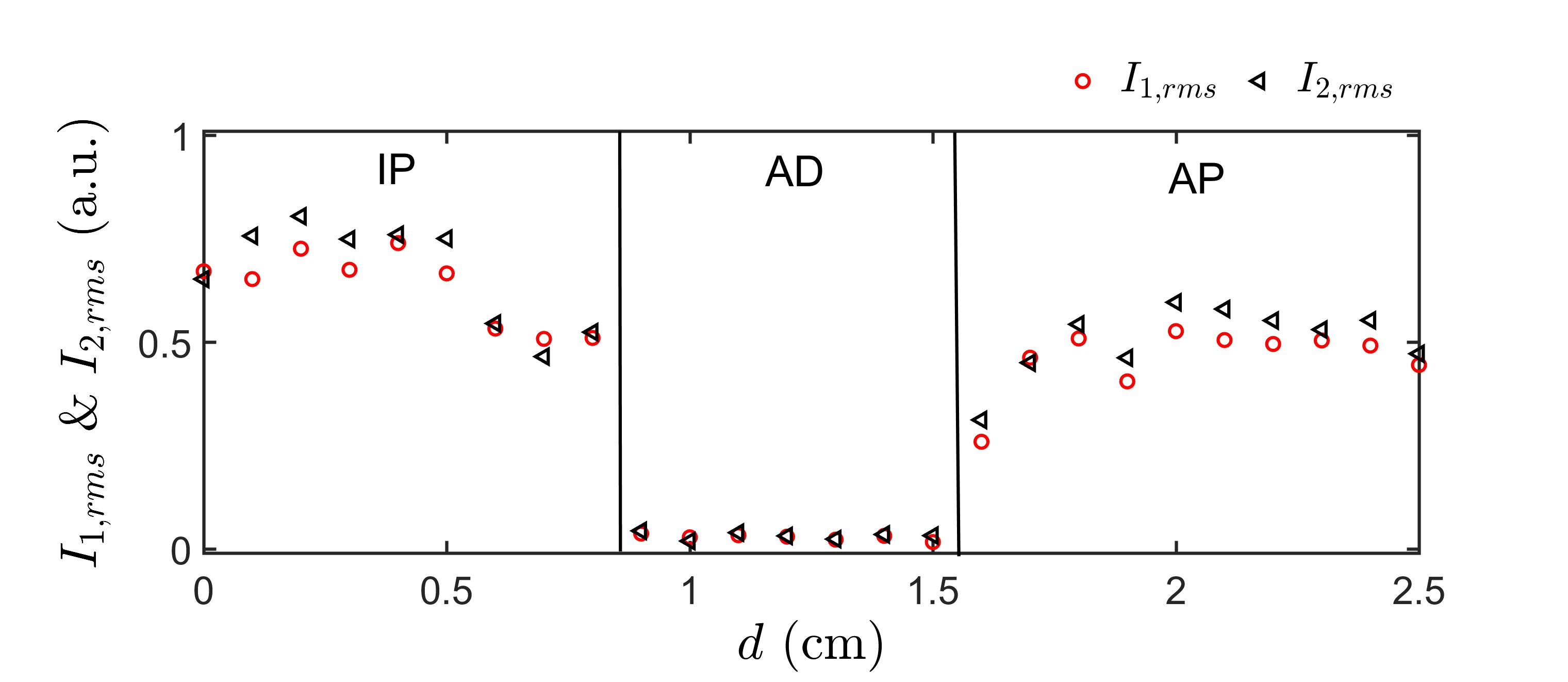


**Supplementary Figure 1: Variation in the root mean square value of the signals obtained from a system of coupled candle-flame oscillators with distance (**$\boldsymbol{d}$**) between them.** The plot highlights the variation of the root mean square value of the signals ($I_{1,rms}$ and $I_{2,rms}$) as the distance between a pair of coupled candle-flame oscillators ($d$) is changed, wherein each oscillator consists of four candles ($N_{c}$ = 4). The plot is divided into three different regions of coupled dynamics depending on the characteristics of the signals observed during these states. Starting from in-phase (IP) mode of oscillation, the root mean square value of each oscillator decreases gradually and eventually reaches a value near zero during the amplitude death (AD) state. When the oscillators regain oscillations in the state of anti-phase (AP) oscillation, after AD, the root mean square value of oscillations begins to increase and reach a nearly constant value. This behaviour of the variation in amplitudes of signals acquired from coupled-candle flame oscillators with distance ($d$) between them is similar when $N_{c}$ equals 3 and 5.

**
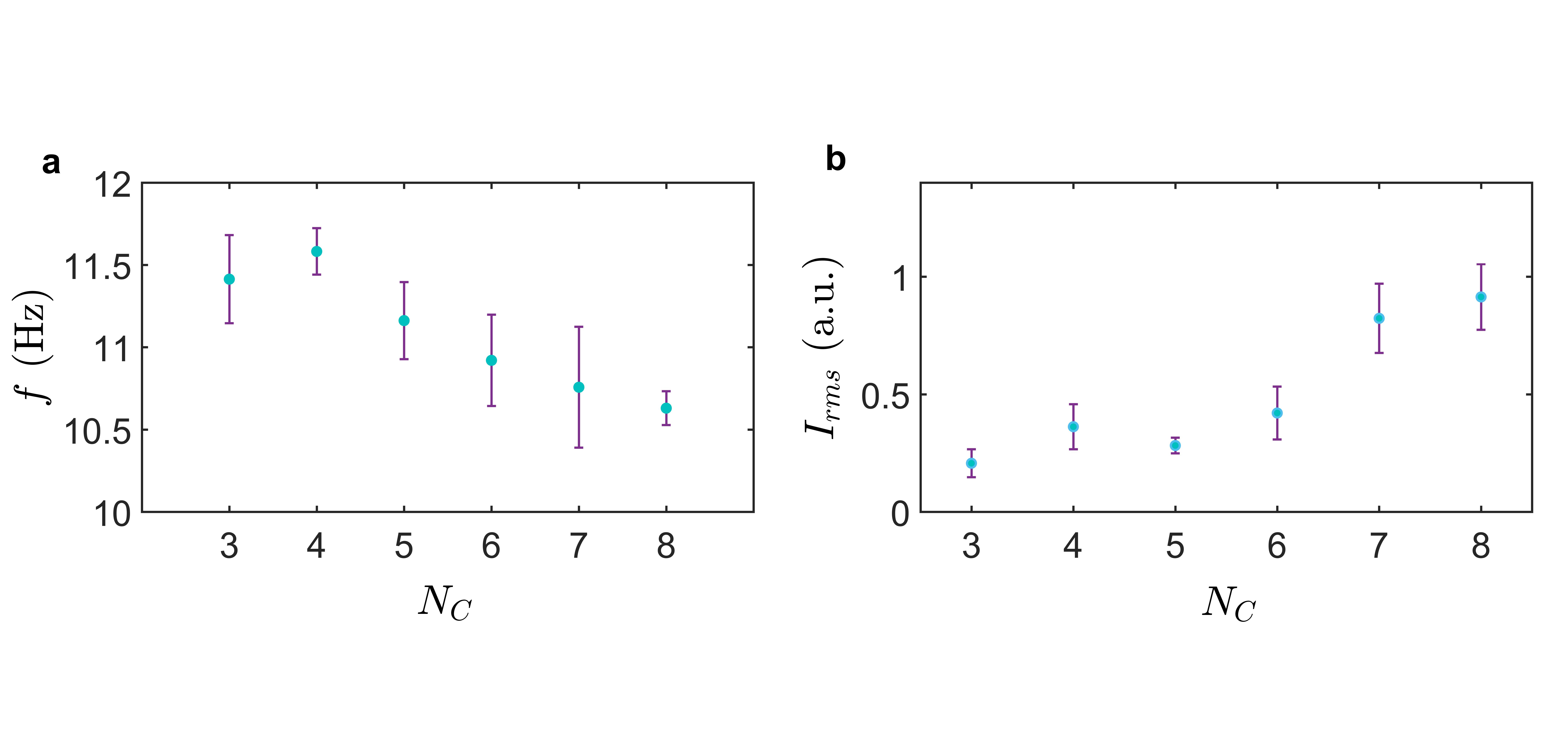
**

**Supplementary Figure 2: Characteristic properties of limit cycle oscillations produced by an isolated candle-flame oscillator with varying number of candles (**$\boldsymbol{N}_{\boldsymbol{C}}$**) in an oscillator.** Variation of **a**, dominant frequency ($f$) and **b**, root mean square value ($I_{rms}$) of self-sustained limit cycle oscillations produced by an isolated candle-flame oscillator when the number of candles ($N_{C}$) in the oscillator is changed. The dominant frequency of oscillations exhibited by such oscillators, obtained from their amplitude spectra, always lie in between 10 Hz to 12 Hz for different values of $N_{C}$ (see **a**). This observation of frequency is in accordance with an important characteristic found in most of the buoyant diffusion flames, that the universal value of the flickering frequency lies in a range of 10-20 Hz, irrespective of the type of system considered^1^. The error bar indicates the ensemble average of frequencies and the root mean square value of the oscillations performed over ten experiments. We also observe a slight decrease in the value of mean of dominant frequency of an oscillator as $N_{C}$ is increased (in **a**). The variation of root mean square value for each candle-flame oscillator exhibits an increasing trend as the number of candles ($N_{C}$) in an oscillator is increased (in **b**). As $N_{C}$ is increased, the rate of fuel supplied to the flame of an oscillator increases. Hence, the oxygen required for complete combustion of the fuel increases. This leads to an increased surface area and volume of the flame, which are essential to satisfy this increased oxygen requirement.

**Supplementary Note 1: Description of time-delay coupled identical Stuart-Landau oscillators:**

We find that the behavior of coupled candle-flame oscillators resembles the behavior exhibited by coupled Stuart-Landau oscillators. Here, the oscillators are identical (that is, the frequency of each oscillator is the same) and are coupled with time delay coupling alone. The general equation for the well-known Stuart-Landau oscillator is given by^2,3^

$\dot{Z}\left( t \right)=\left( a+i\omega-\left| Z\left( t \right) \right|^{2} \right) Z(t)$ (1)

where $Z\left( t \right)$ is a complex variable given as $Z\left( t \right)=\sqrt{a}e^{i\omega t}$, $\sqrt{a}$ being the amplitude of the oscillator ($a$ > 0) and $\omega$ being the natural frequency of oscillation. We choose the value of *a* (= 1) such that each oscillator exhibits a stable limit cycle oscillation in an uncoupled state.

Therefore, the equations for the linearly coupled Stuart-Landau oscillators with time delay coupling are given by

$\dot{Z_{1}}\left( t \right)= [1+i\omega_{1}-\left| Z_{1}\left( t \right) \right|^{2}]Z_{1}\left( t \right)+K[Z_{2}\left( t-\tau\right)-Z_{1}\left( t \right)]$ (2)

$\dot{Z_{2}}\left( t \right)= [1+i\omega_{2}-\left| Z_{2}\left( t \right) \right|^{2}]Z_{2}\left( t \right)+K[Z_{1}\left( t-\tau\right)-Z_{2}\left( t \right)]$ (3)

where the subscripts 1 and 2 correspond to each Stuart-Landau oscillator. The second term on the right-hand side in both the equations (2) and (3), for instance $K[Z_{2}\left( t-\tau\right)-Z_{1}\left( t \right)]$, contributes to the time delay coupling. In the equations, $\tau$ corresponds to the time delay and $K$corresponds to the coupling strength between the oscillators.

We notice that the behaviour observed at low and high values of $N_{C}$ (number of candles in an oscillator) with increasing $d$ (distance between the oscillators) in a system of coupled candle-flame oscillators is qualitatively similar to that observed at respective low and high values of $K$ (coupling strength) with increasing $\tau$ (time delay) in the model of coupled Stuart-Landau oscillators. Further, we notice in experiments that with increase in $N_{C}$, the amplitude of oscillations of an individual candle-flame oscillator increases (see Supplementary Fig. 2b) and, we conjecture that this increase in amplitude contributes to an increase in the coupling strength between the oscillators. Further, we consider the oscillators as identical ($\omega_{1}=\omega_{2}=\omega$), as the natural frequency of a pair of candle-flame oscillators in their uncoupled state is nearly equal, when $N_{c}$ is the same in both the oscillators. The results of coupled Stuart-Landau oscillators for the transition from in-phase (IP) to anti-phase (AP) through the amplitude death (AD) state, and the direct transition through phase-flip bifurcation (PFB) are shown in Supplementary Figs. 3 and 4, respectively. A two-parameter plot showing the variation of $K$ with $\tau$ for these oscillators is shown in Supplementary Fig. 5.

The initial conditions of $Z_{1}$ and $Z_{2}$ are fixed at 0.3 and 0.5, respectively, and the non-dimensional frequencies ($\omega$) of both oscillators are kept the same at 10 throughout the study. Data was obtained for 200 units with the transient of first 50 units ignored from the signals.

**
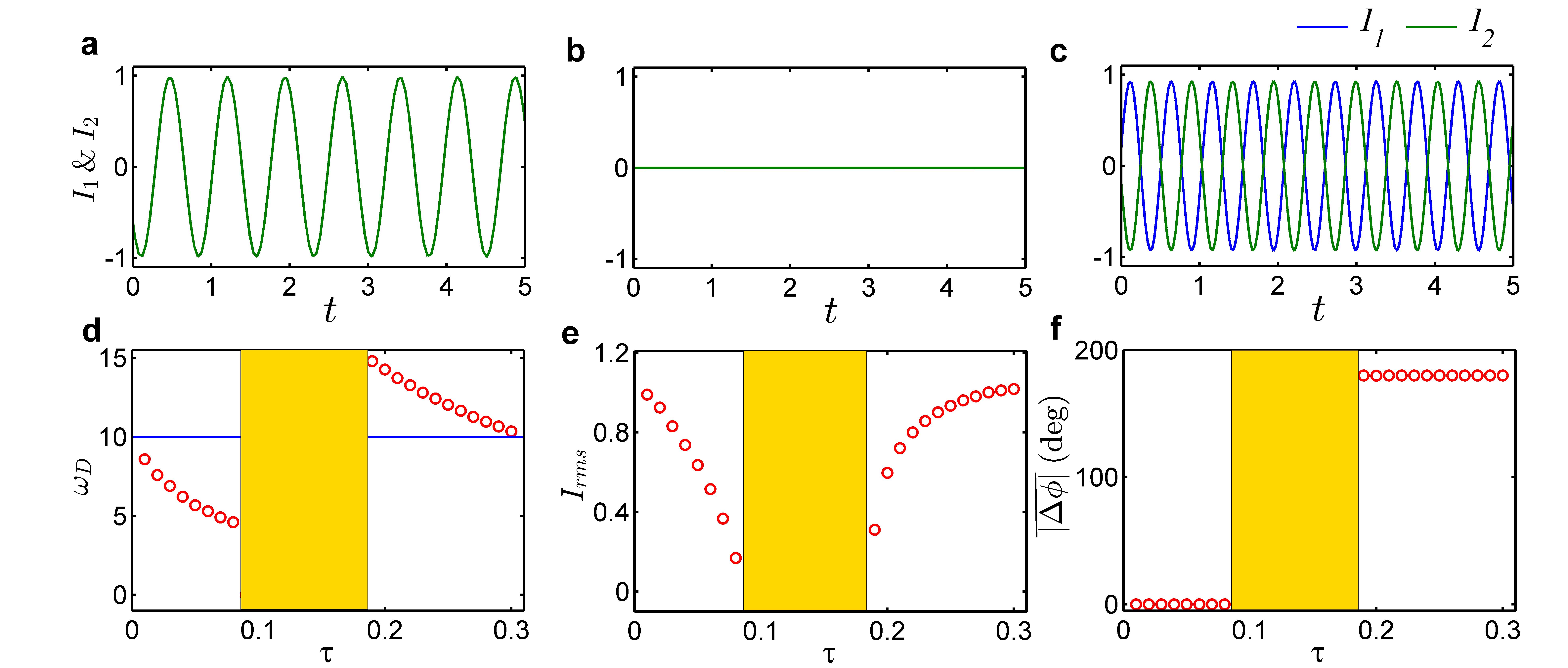
**

**Supplementary Figure 3: Synchronization transition from in-phase (IP) to anti-phase (AP) state via intermediate amplitude death (AD) state for coupled identical Stuart-Landau oscillators. a-c,** The times series of IP, AD and AP states observed for coupled Stuart-Landau oscillators for different values of time delay ($\tau$) such as 0, 0.15 and 0.25, respectively, at a constant value of coupling strength ($K$) equal to 15. **d-f,** The variation of the dominant frequency, the root mean square value of amplitude of oscillations and the mean phase difference between the oscillators for different values of $\tau$. For a constant low value of $K$(= 15), we observe that as $\tau$ is increased, both oscillators transition from IP to AP states of oscillation via an intermediate state of AD. During IP state, the frequency of coupled oscillators is observed to decrease from the frequency of an uncoupled oscillator ($\omega$= 10, shown by horizontal line in **d**), whereas it shows a significant jump during the onset of AP, which eventually decreases and approaches a value close to the frequency of an uncoupled oscillator. The response amplitude of coupled oscillators (in **e**) shows a continuous decrease and increase during IP and AP states, respectively. However, the amplitude of oscillations is nearly zero, showing the cessation of oscillations, during AD state. The mean phase difference between the signals of both oscillators shows a value of zero deg during IP state and a value of 180 deg during AP state (in **f**). Such variation in the properties of coupled Stuart-Landau oscillators due to the change in delay between the oscillators for a constant value of the coupling strength are qualitatively similar to that observed in the experiments of coupled candle-flame oscillators (refer Figs. 2 and 3a in main text and Supplementary Fig. 1), when the number of candles in an oscillator were low (3 to 5). This supports our conjecture of the importance of time delay in displaying AD state in between the states of IP and AP synchronization in a pair of coupled candle-flame oscillators.

**
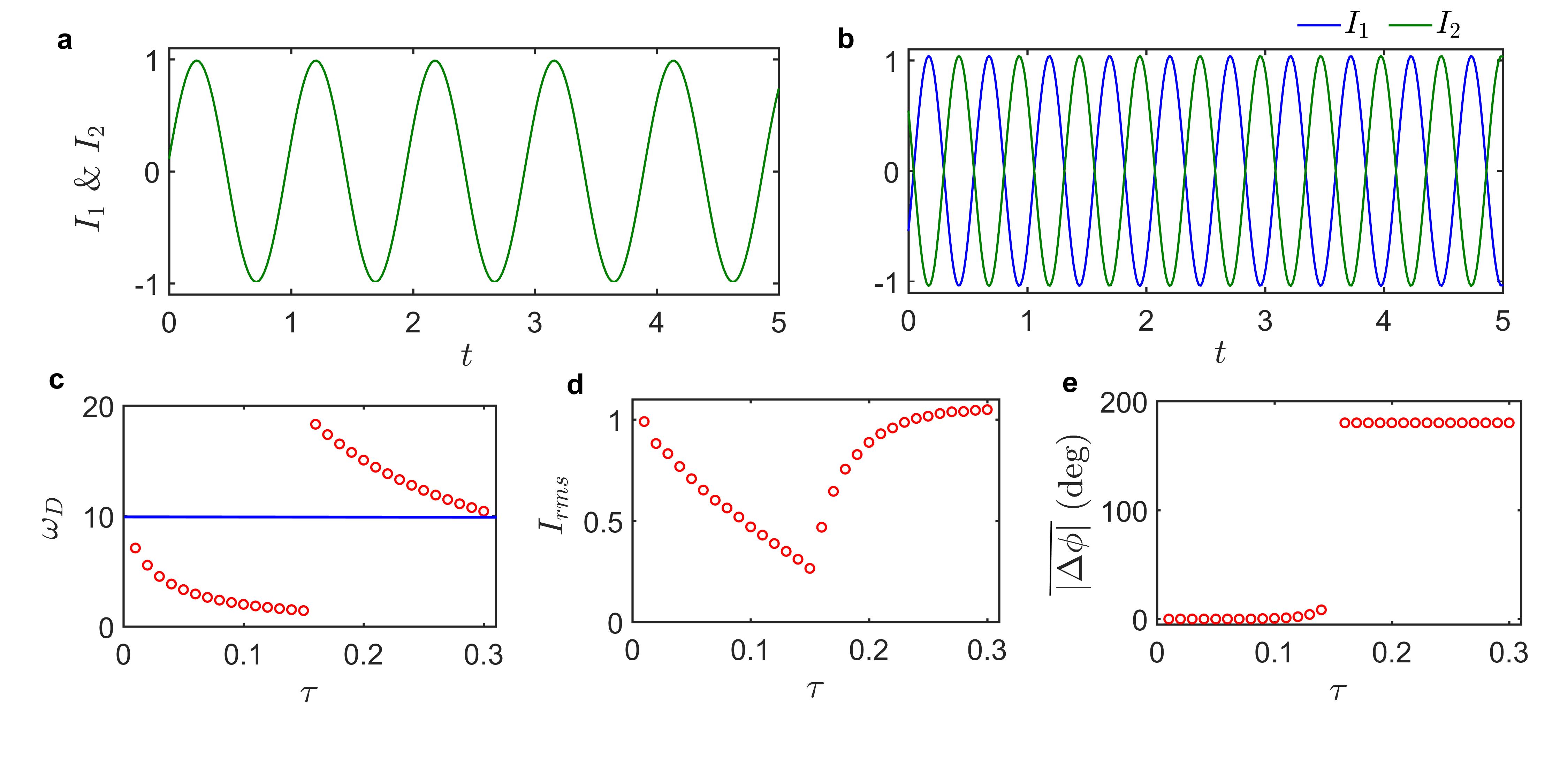
**

**Supplementary Figure 4: Synchronization transition from in-phase (IP) to anti-phase (AP) mode through phase-flip bifurcation (PFB) for coupled identical Stuart-Landau oscillators.** The time series corresponding to **a,** IP and **b,** AP states of oscillations for a constant higher value of coupling strength ($K$ = 55) obtained at different values of time delay ($\tau$) as 0.01 and 0.25, respectively. **c-e,** The variation of the dominant frequency, the root mean square amplitude and the mean phase difference between the oscillators with $\tau$. When the value of $K$(= 55) is considerably high, we observe a sudden transition from IP to AP state. This abrupt transition is accompanied by an equivalent jump in the value of the dominant frequency and the mean relative phase between the oscillators. In contrast, the amplitude of oscillations is observed to decrease and then increase gradually during the transition from IP to AP states. At this value of $K$, we observe the exhibition of PFB by coupled Stuart-Landau oscillators as a result of varying time delay between them. These results are qualitatively similar to that observed for coupled candle-flame oscillators consisting of eight candles in each oscillator (refer Figs. 4b,c in main text).


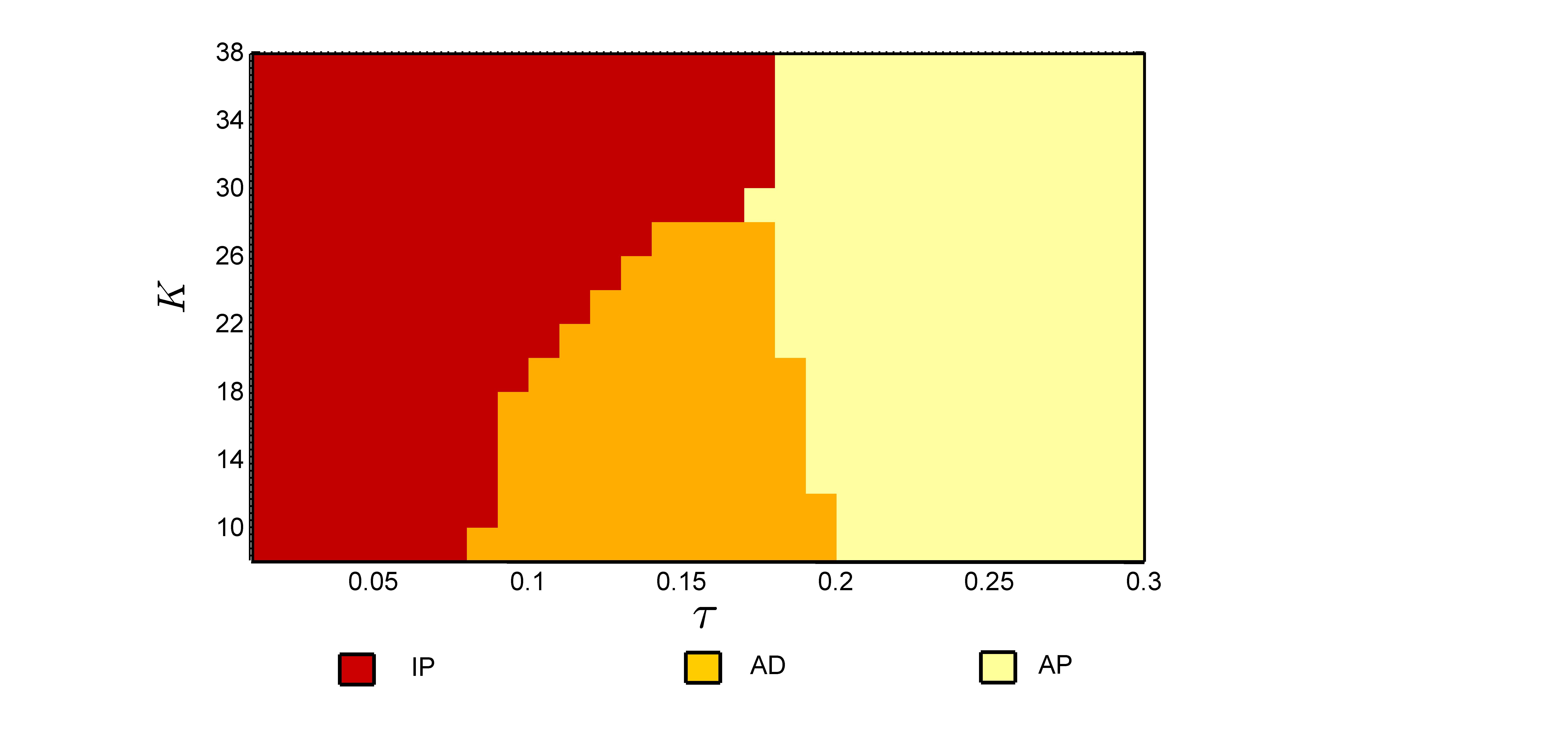


**Supplementary Figure 5: Two-parameter bifurcation plot between time delay (**$\boldsymbol{\tau}$**) and coupling strength (**$\boldsymbol{K}$**) for a system of time-delay coupled identical Stuart-Landau oscillators.** Figure shows the mapping of different dynamical states such as in-phase (IP), amplitude death (AD) and anti-phase (AP) in a parameter space of $K$ and $\tau$. At low values of $K$, the transition from IP to AP state happens via the AD state (refer Supplementary Fig. 3), whose region gradually decreases as $K$ is increased. Further, a sufficient increase in $K$ value causes the complete disappearance of the AD zone and the emergence of a new phenomenon of phase-flip bifurcation (PFB) (refer Supplementary Fig. 4). During PFB, the oscillators shift their coupled dynamics directly from IP to AP oscillations accompanied by a jump in the value of frequency and phase difference. Thus, the presence of time delay alone in a system of identical oscillators (oscillators with the same natural frequency) can result in the coexistence of two different phenomena such as AD and PFB. These results are very much similar to Fig. 4 in main text, where the distance between the coupled candle-flame oscillators contributes to the delay in their coupling and the number of candles contributes to the change in coupling strength between the oscillators.


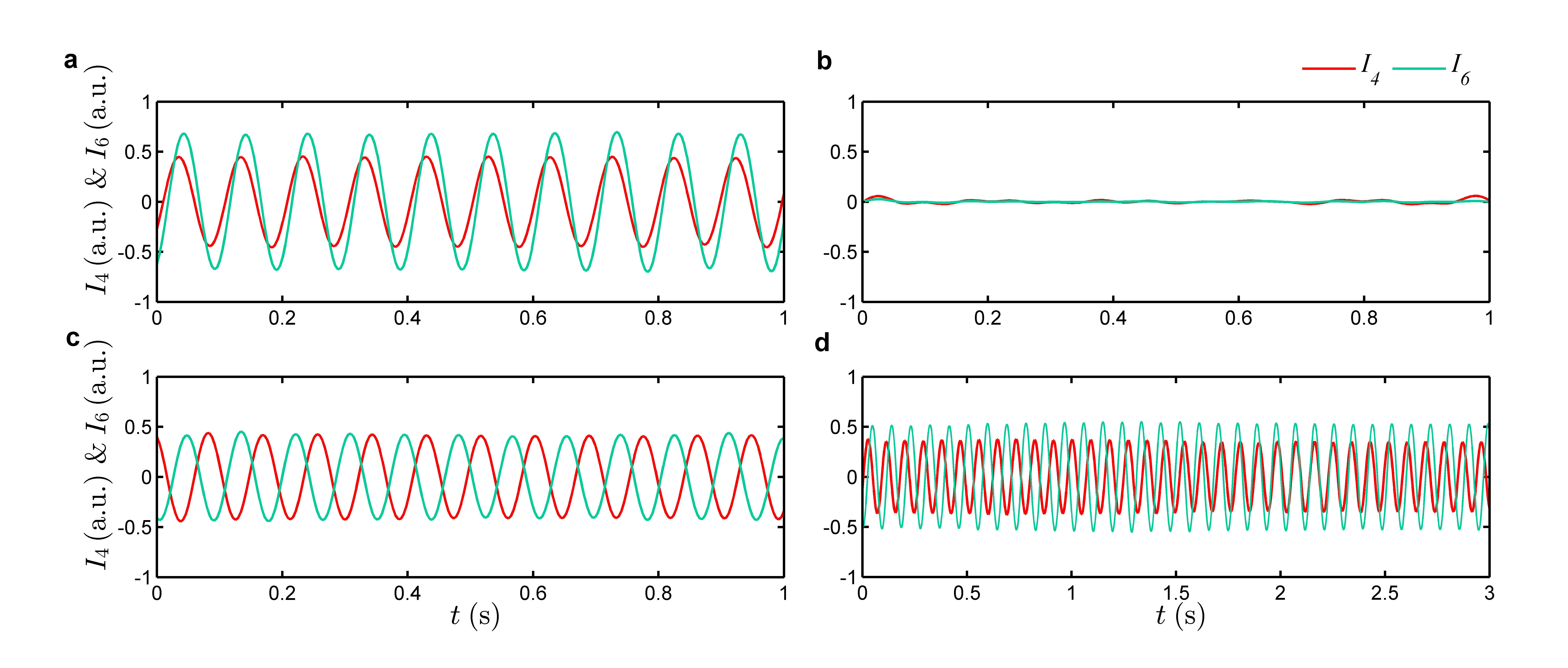


**Supplementary Figure 6: Various modes of coupled dynamics observed in experiments of a dissimilar (**$\boldsymbol{N}_{\boldsymbol{c}}$ **is not same) pair of candle-flame oscillators.** **a-d**, The temporal variation of heat release rate fluctuations corresponding to in-phase (IP), amplitude death (AD), anti-phase (AP) and desynchronized states of oscillations, respectively, for a coupled dissimilar pair (that is, number of candles in each oscillator is different) of candle-flame oscillators (see Supplementary Videos 8-11). The instantaneous value of the heat release rate fluctuations, $I_{4}$ corresponds to the oscillator consisting of 4 candles mounted on the movable platform, and $I_{6}$ corresponds to oscillator with 6 candles mounted on the stationary platform. These states are observed at different distances ($d$) between the oscillators of 0 cm, 1.2 cm, 2 cm and 7.5 cm, respectively. We observe that the flame dynamics observed at various values of $d$ in a dissimilar pair of oscillators is very much akin to that observed in a similar pair (that is, same number of candles in both oscillators) (refer Fig. 2 in main text). The amplitude of oscillation is observed to be different for each oscillator, which is due to the difference in the amplitudes of their individual oscillations (see Supplementary Fig. 2b).


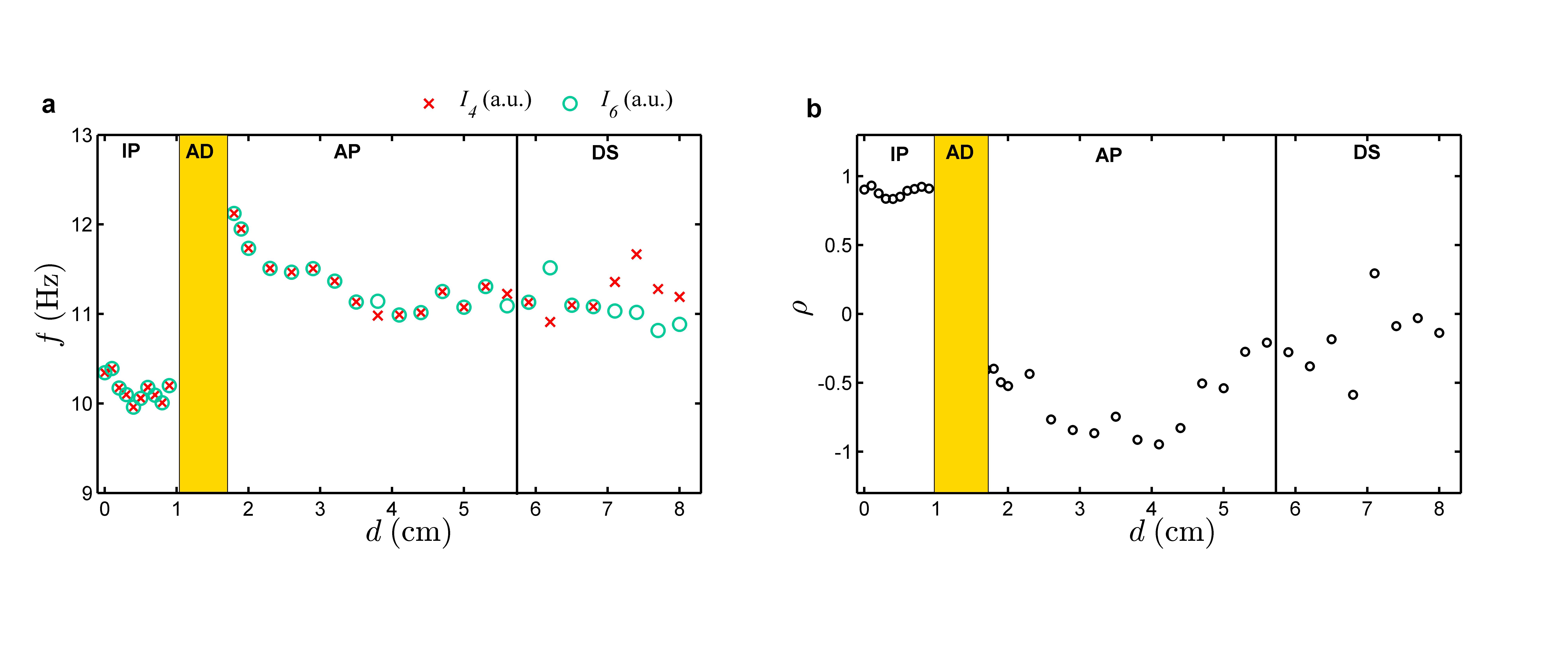


**Supplementary Figure 7:** **Dependence of dominant frequency (**$\boldsymbol{f}$**) and Pearson’s correlation coefficient (**$\boldsymbol{\rho}$**) on the distance between the oscillators (**$\boldsymbol{d}$**) for a dissimilar pair of candle-flame oscillators.** Variation in **a,** the dominant frequency ($f$) and **b,** the Pearson’s correlation coefficient ($\rho$) with $d$ for a coupled dissimilar pair of candle-flame oscillators. During in-phase (IP), we observe a drop in the frequency of both the oscillators compared to their uncoupled frequency value along with a near positive one value of $\rho$. Whereas, during the onset of anti-phase (AP), the frequency exhibits a significant rise accompanied with the value of $\rho$ near negative one. During the desynchronized state (DS), the frequency of the coupled oscillators fluctuates around their individual isolated frequencies and the value of $\rho$ tends to zero. The presence of amplitude death (AD) region observed in between the IP and AP states is highlighted in both **a** and **b**. Hence, we conclude that the dynamic transition of coupled dissimilar candle-flame oscillators is near identical to that of similar candle-flame oscillators (see Fig. 3 in main text), except for the presence of transition states.

**References**

1. Buckmaster J. & Peters N. The infinite candle and its stability-a paradigm for flickering diffusion flames. *Proc. Combust. Inst.* **21**, 1829-1836 (1988).
2. Reddy, D. R., Sen, A. & Johnston, G. L. Time delay induced death in coupled limit cycle oscillators. *Phys. Rev. Lett*.**80,** 5109 (1998).
3. Atay, F. M. ed., *Complex Time-Delay Systems: Theory and Applications* (Springer, 2010).
